# Association Study of the Complement Component C4 Gene in Tardive Dyskinesia

**DOI:** 10.3389/fphar.2019.01339

**Published:** 2019-11-26

**Authors:** Clement C. Zai, Arun K. Tiwari, Gwyneth C. Zai, Natalie Freeman, Jennie G. Pouget, James Greco, Maria Tampakeras, Sajid A. Shaikh, Deanna Herbert, Heather Emmerson, Sheraz Y. Cheema, Nicole Braganza, Daniel J. Müller, Aristotle N. Voineskos, Gary Remington, James L. Kennedy

**Affiliations:** ^1^Tanenbaum Centre for Pharmacogenetics, Campbell Family Mental Health Research Institute, Centre for Addiction and Mental Health, Toronto, ON, Canada; ^2^Department of Psychiatry, University of Toronto, Toronto, ON, Canada; ^3^Institute of Medical Science, University of Toronto, Toronto, ON, Canada; ^4^Laboratory Medicine and Pathobiology, University of Toronto, Toronto, ON, Canada

**Keywords:** pharmacogenetics, tardive dyskinesia (TD), complement component *C4*, schizophrenia, antipsychotic

## Abstract

Tardive dyskinesia (TD) is a movement disorder that may develop in schizophrenia patients being treated long-term with antipsychotic medication. TD interferes with voluntary movements and leads to stigma, and can be associated with treatment non-adherence. The etiology of TD is unclear, but it appears to have a genetic component. There is emerging evidence of immune dysregulation in TD. In the current study, we set out to investigate the complex schizophrenia-associated complement component 4 (*C4*) gene for possible association with TD occurrence and TD severity as assessed by the Abnormal Involuntary Movement Scale (AIMS) in a sample of 129 schizophrenia patients of European ancestry. We have genotyped the copy numbers of long and short forms of *C4*A and *C4*B gene variants in 129 European ancestry patients with schizophrenia or schizoaffective disorder. We did not find predicted *C4*A or *C4*B expression to be nominally associated with TD risk or severity. However, we found the number of copies of *C4*BL to be nominally associated with TD severity (*p* = 0.020).

## Introduction

Schizophrenia is a chronic and severe psychiatric disorder characterized by, amongst other symptoms, hallucinations and delusions. Its etiology is complex and remains unclear, but immune-related mechanisms have been postulated (Khandaker and Dantzer, 2016; van Kesteren et al., 2017). In 2009, two genome-wide association studies implicated the major histocompatibility complex (MHC) region in schizophrenia (Shi et al., 2009; Stefansson et al., 2009). Symptoms of schizophrenia are treated with antipsychotics, which, as a medication class, is associated with risk of developing tardive dyskinesia (TD). The etiology of TD remains unclear (Lee and Kang, 2011; Zai et al., 2018a; Zai et al., 2018b), but a genetic component has been supported by family studies (Weinhold et al., 1981; Yassa and Ananth, 1981; Müller et al., 2001).

The immune system has not been extensively studied in TD. One study reported serum interleukin-2 receptor (IL2R) levels to be increased in antipsychotic-naive or typical antipsychotic-medicated schizophrenia patients with TD *versus* those without (Rapaport and Lohr, 1994), while another study reported IL2 levels to be decreased in chronic-medicated schizophrenia patients with TD compared to those without (Liu et al., 2012). IL2 serum levels have also been correlated positively to TD severity as measured by the Abnormal Involuntary Movement Scale (AIMS) (An et al., 2015). In yet another such study, IL6 levels were found decreased in schizophrenia patients with TD compared to those without (An et al., 2015). Along similar lines, marginally decreased serum TNF-alpha levels were found in medicated chronic schizophrenia patients with TD *versus* those without (Tian et al., 2014). The *TNF* marker -308A/G (rs1800629) has been found to be associated with TD severity (Wang et al., 2012), whereas the *IL10* markers rs1800872 and rs72393728 were not significantly associated with TD in another study (Sun et al., 2013).

The complement component 4 (C4) mediates immune function within the complement system, which is involved in the body’s defense against foreign pathogens. The system consists of the classical, lectin, and alternative pathways that converge on the cleavage of C3 into its active fragments C3a and C3b, resulting in phagocytosis, inflammation, and cell lysis by formation of the membrane attack complex (reviewed in Woo et al., 2019 In Huntington’s disease patients who often manifest dyskinesia reminiscent of TD, complement components of the classical immune pathway, including C4, were found in their striatum, but not so in control brains (Singhrao et al., 1999). Increased levels of complement pathway components including C4 have been reported in serum and cerebrospinal fluids of neurodegenerative diseases such as amyotrophic lateral sclerosis (Bahia El Idrissi et al., 2016). Furthermore, *C4* deficiency has been shown to protect against post-injury motor deficits and brain tissue damage in the controlled cortical impact mouse model of traumatic brain injury (You et al., 2007). Of note, while the *C4* gene has been implicated in schizophrenia (Sekar et al., 2016), it has not been investigated in TD. The *C4* gene (localized to the MHC class III region on 6p) has a complex structure. An individual may have different copy numbers of two possible isotypes of *C4*, *C4*A and *C4*B, which have different molecular targets (Sekar et al., 2016). *C4*A and *C4*B also exists as either a short (S) form or long (L) form with the insertion of a human endogenous retrovirus in intron 9 (Wu et al., 2007), resulting in four potential compound structural forms of *C4*: *C4*AL, *C4*AS, *C4*BL, and *C4*BS. The insertion of the human endogenous retrovirus has been shown to downregulate the expression of the parent *C4* gene (Yang et al., 2003; Mack et al., 2004).

## Methods

### Subjects

For the current study, we included 87 participants for which the sample characteristics have been described previously (GR; Zai et al., 2007; Zai et al., 2013). Briefly, participants were enrolled from the Center for Addiction and Mental Health in Toronto, Ontario (Dr. G Remington), and were of European ancestry. Participants were diagnosed with schizophrenia or schizoaffective disorder based on DSM-III-R or DSM-IV criteria (APA, 2000 #3). These chronic patients had been exposed to typical antipsychotic medication for at least 1 year before TD assessment. Exclusion criteria included type II diabetes, head injury with loss of consciousness, and seizure disorder. TD occurrence was classified based on the Schooler and Kane criteria using the AIMS (Schooler and Kane, 1982), with individuals having at least one moderate rating or at least two mild ratings on the first seven items in the AIMS being categorized as having probable TD (Schooler and Kane, 1982). Our sample has over 80% power to detect an effect size (variance explained Rg^2^) of 0.06 [*α* = 0.05, Quanto v1.2.3; (Gauderman and Morrison, 2006)]. In addition, we included an independent sample of 49 participants from the IMPACT (The Individualized Medicine: Pharmacogenetics Assessment and Clinical Treatment) naturalistic pharmacogenetic study. These participants were of self-reported European ancestry who were clinically diagnosed with schizophrenia or schizoaffective disorder and assessed for tardive dyskinesia (Zai et al., 2018c #574; Herbert, 2018 #470). Total AIMS scores were available for all 136 patients. We obtained informed consent from each participant enrolled and the study was approved by the CAMH research ethics board.

### Genetic Data Collection

Genomic DNA was extracted from whole blood using high-salt method (Lahiri and Nurnberger, 1991) or extracted from saliva (DNA Genotek) using automated DNA extraction (Chemagen). In the Sekar et al. (2016) paper, the authors used droplet digital PCR (ddPCR) to determine the precise copy numbers of *C4*A, *C4*B, *C4*L, and *C4*S, with RPP30 as the diploid control. Then, selecting for individuals with at least one copy of C4S, they amplified the *C4*S using long-range PCR, and by using ddPCR, determine the number of *C4*AS and *C4*BS, and thereafter infer the copy number of *C4*AL and *C4*BL. We used a similar approach to determine the number of *C4*AL, *C4*BL, *C4*AS, and *C4*BS for our study. Copy number assays for the four structural elements [*C4*A (Hs07226349_cn), *C4*B (Hs07226350_cn), *C4*L (Hs07226352_cn), and *C4*S (Hs07226351_cn)] were run on Viia 7 Real-time PCR System (Thermo Fisher Scientific) in quadruplicates with RNaseP reference assay following manufacturer’s protocol, and the copy numbers of *C4*A, *C4*B, *C4*L, and *C4*S were determined using the CopyCaller software (Thermo Fisher Scientific). For individuals with at least one copy of *C4*S, we followed up by running long-range PCR with standard protocol using the following primers specific for *C4*S: forward 5′-TCAGCATGTACAGACAGGAATACA-3′ and reverse 5′-GAGTGCCACAGTCTCATCATTG-3′ (TaKaRa, Clontech) (Sekar et al., 2016). We subsequently ran a TaqMan-based genotyping assay to determine the presence of *C4*A and/or *C4*B within the *C4*S long-range PCR product (Thermo Fisher Scientific). From this follow-up step, we determined the number of *C4*AS and *C4*BS in our sample, and then deduced the number of *C4*AL and *C4*BL by subtracting from the total numbers of *C4*A, *C4*B, *C4*L, and *C4*S. Genotype quality was checked by the formula [*C4*A + *C4*B = *C4*L + *C4*S] and the presence of long-range PCR product in individuals with at least one copy of *C4*S. We re-genotyped the samples where the numbers did not match and could not resolve seven samples, which we excluded from the analyses. More information on the two samples is shown in **Table 1**.

**Table 1:** Characteristics of the samples analyzed in this study.

Characteristics	GR	IMPACT
Sex (Male/Female)	51/32	35/11
Age (mean ± SD)	42.34 ± 9.84	40.17 ± 12.32
TD occurrence (Yes/No)	32/51	16/30
Total AIMS scores (mean ± SD)*	6.01 ± 6.74	4.11 ± 4.62
*C4*A copy number	2.07 ± 0.66	2.02 ± 0.58
*C4*B copy number	1.88 ± 0.59	1.85 ± 0.36
*C4*L copy number	3.02 ± 0.95	3.17 ± 0.93
*C4*S copy number	0.93 ± 0.87	0.70 ± 0.66
*C4*AL copy number	1.96 ± 0.65	1.98 ± 0.54
*C4*BL copy number	1.06 ± 0.77	1.20 ± 0.69
*C4*AS copy number	0.11 ± 0.31	0.04 ± 0.21
*C4*BS copy number	0.82 ± 0.83	0.65 ± 0.64
*C4*A expression	1.19 ± 0.33	1.19 ± 0.32
*C4*B expression	1.81 ± 0.57	1.81 ± 0.37

* Significant difference between GR and IMPACT samples, with p = 0.014.

### Statistical Analysis

Sex ratio, average age, average total AIMS scores, and TD occurrence rate did not differ between the two samples (*p* > 0.05). Thus, we conducted statistical analyses on the pooled dataset. TD occurrence was analyzed with sex using Pearson chi-squared test. The rate of TD was not significantly different between males (34.65%) and females (43.75%) in the collective sample (*p* > 0.05). AIMS scores were not normally distributed; thus, we performed log transformation. Log-transformed AIMS scores were compared between males and females using Student’s *t* test, where the findings were not significant (*p* > 0.05). However, significant positive correlation was found between AIMS scores and age (*p* < 0.001). Further, average age was compared between TD-positive and TD-negative groups using Student’s *t* test and was significantly higher in the TD-positive group than the TD-negative group (*p* < 0.001). The analyses of TD occurrence with the numbers of structural elements (*C4*A, *C4*B, *C4*L, and *C4*S) as well as with the numbers of compound structural forms (*C4*AL, *C4*AS, *C4*BL, and *C4*BS) were performed using logistic regression, with age, sex, and sample set included as covariates. The analyses of log-transformed AIMS scores were carried out using linear regression, with age, sex, and sample set as covariates. We used the following formulas provided by Sekar et al. to predict brain expression (composited from expression measures in up to five brain regions from three postmortem brain samples; Sekar et al., 2016) of *C4*A and *C4*B: [*C4*A expression = (0.47 x *C4*AL) + (0.47 x *C4*AS) + (0.20 x *C4*BL)] and [*C4*B expression = (1.03 x *C4*BL) + (0.88 x *C4*BS)]. The predicted expression levels were analyzed with TD occurrence using logistic regression and log-transformed AIMS scores using linear regression.

## Results

**Figure 1:**
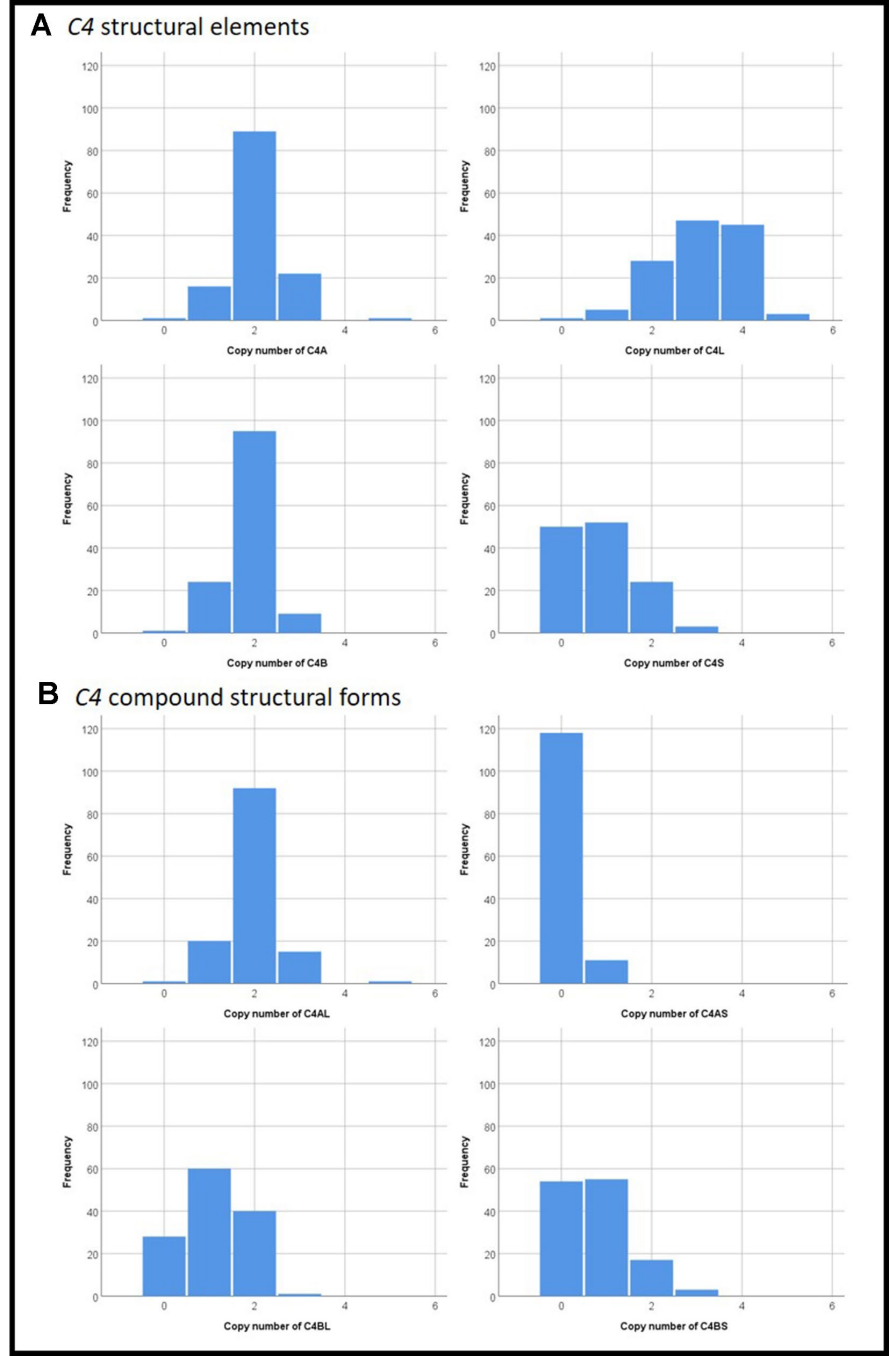
Distributions of copy numbers of the various *C4* **(A)** structural elements and **(B)** compound structural forms in our study sample.

**Figure 2:**
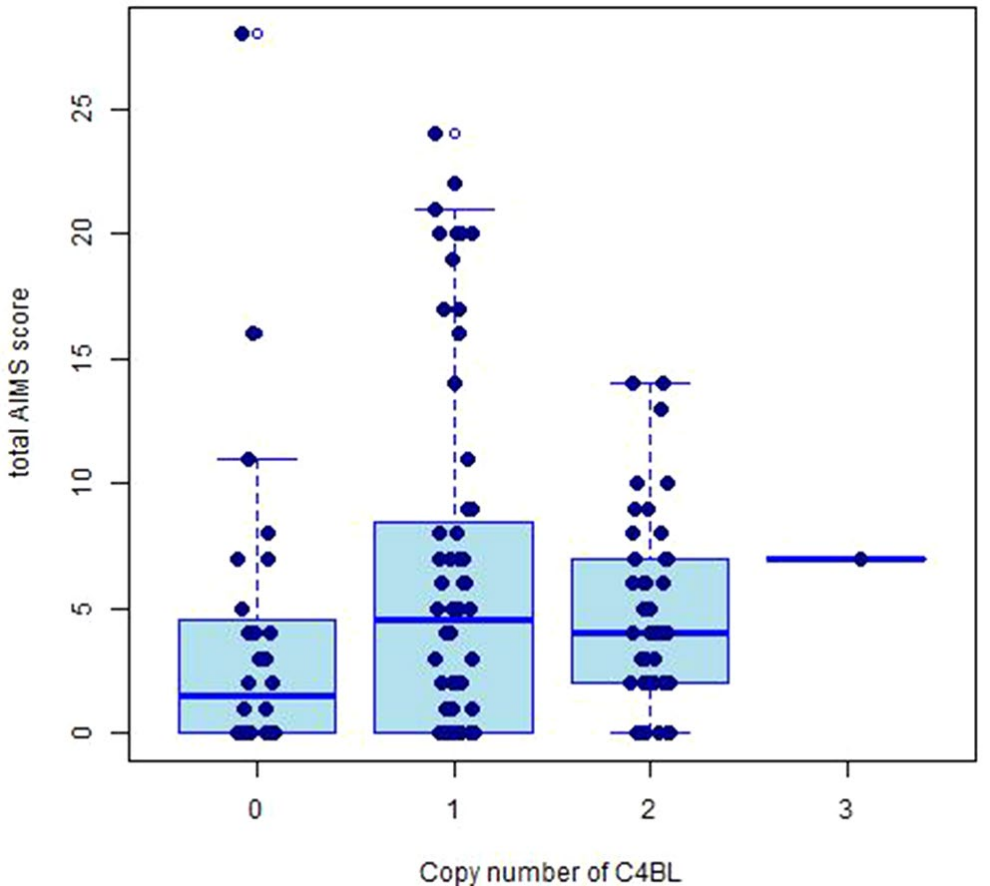
Box plot displaying the distributions of total AIMS scores for copy numbers of *C4*BL.

The copy number distributions of *C4* structural variants in our samples are shown in **Figure 1**. The copy numbers of structural elements (*C4*A, *C4*B, *C4*L, *C4*S) and compound structural forms (*C4*AL, *C4*AS, and *C4*BS) were not associated with TD occurrence or TD severity in our pooled sample (*p* > 0.05). We observed a nominally significant association of *C4*BL with TD severity (*B*(±SE) = 0.118 ± 0.05; *t* = 2.36, *p* = 0.020; **Figure 2**). Neither estimated *C4*A nor *C4*B expression was associated with TD occurrence or TD severity (*p* > 0.05).

## Discussion

This is the first study to examine the *C4* gene in TD, and we found a possible association between *C4*BL copy number and TD severity. However, it is important to note that due to the exploratory nature of our study, we did not correct for multiple testing, and our findings with copy number of *C4*BL would not have survived Bonferroni correction.

While previous studies have implicated low copy number of *C4*A (Li et al., 2017; Juptner et al., 2018) in autoimmune diseases, including systematic lupus erythematosus and rheumatoid arthritis, increased *C4*A was associated with schizophrenia susceptibility, possibly through its role in synaptic pruning (Sekar et al., 2016). The role of *C4*B in autoimmune diseases and schizophrenia needs further studies (Li et al., 2017), but low *C4*B copy number has been implicated in cardiovascular disease risk (Blasko et al., 2008). While C4A targets amino-group containing antigens, C4B preferentially targets carbohydrate antigens (Blanchong et al., 2001). One potential hypothesis for the observed association here is that disrupted sphingolipid metabolism by high-TD-risk antipsychotics such as haloperidol (McClay et al., 2015) may trigger *C4*B to mediate the white matter disruption observed in TD patients (Bai et al., 2009). Interestingly, a genome-wide association study has implicated the proteolipid protein 1 (*PLP1*) gene in severity of extrapyramidal side effect as measured by the Simpson–Angus Scale (Aberg et al., 2010). Further studies of white matter integrity in TD patients may be warranted.

In addition, complement component *C3* has been implicated in metabolic syndrome in schizophrenia patients treated with clozapine (reviewed in Woo et al., 2019) Genes coding for other members in the complement systems that have been associated with schizophrenia susceptibility (Schizophrenia Working Group of the Psychiatric Genomics Consortium, 2014), including the CUB and Sushi multiple domains 1 (*CSMD1*), *C2*, and Complement Factor B (*CFB*), may be an avenue for future genetic investigations of the complement system in TD.

Our study has a number of limitations. In addition to structural elements and compound structural forms examined in this study, the compound structural forms may be configured in various combinations, or structural haplotypes, which will require additional molecular analyses and parental genotypes to resolve. Thus, we were unable to determine the *C4* structural haplotypes with our experimental approach and we were not able to resolve the copy number of *C4*AL, *C4*BL, *C4*AS, or *C4*BS in about 1% of the sample using our experimental workflow. Our sample was of moderate size; thus, while our suggestive findings with *C4*BL may be due to chance, our lack of significant findings with *C4*A expression and *C4*B expression could be due to insufficient sample size. We also do not have detailed information on clinical variables that could have influenced the findings. These variables include age at onset of TD, treatment duration before TD onset. Thus, further studies in larger samples are required before these findings can be firmly established. Overall, our preliminary findings encourage additional studies of the *C4* gene in schizophrenia-related phenotypes including antipsychotic medication response and side effects.

## Data Availability Statement

The datasets for this manuscript are not publicly available because of issues of data ownership and participant consent. Requests to access the datasets should be directed to Dr. James L. Kennedy, jim.kennedy@camh.ca, or Dr. Clement C. Zai, clement.zai@camh.ca.

## Ethics Statement

The studies involving human participants were reviewed and approved by CAMH Research Ethics Board. The patients/participants provided their written informed consent to participate in this study.

## Author Contributions

CZ and NF oversaw the overall genetic study design. DH, HE, SC, NB, AV, and GR were involved in participant recruitment. SS and MT prepared the DNA samples. CZ, JG, and MT genotyped the samples. CZ and AT conducted the statistical analyses. CZ prepared the first draft of the manuscript. GZ, JP, AT, DM, GR, and JK reviewed the manuscript.

## Funding

CZ, AT, and JK were supported by Genome Canada Genomic Applications Partnership Program (GAPP) and the CAMH Foundation. DM is supported by the Canadian Institutes of Health Research (CIHR Operating Grant MOP 142192), the National Institutes of Health (R01MH085801), the Centre for Addiction and Mental Health Foundation (Joanne Murphy Professorship) and received a Brain & Behaviour Research (NARSAD) Independent Investigator Award, the Michael Smith New Investigator Salary Prize for Research in Schizophrenia (CIHR) and an Early Researcher Award by the Ministry of Research and Innovation of Ontario. GR is supported by the Canadian Institutes of Health Research (CIHR), as well as the Research Hospital Fund - Canadian Foundation for Innovation (RHF-CFI). We would also like to express gratitude towards Larry and Judy Tanenbaum for their generous support in creating the Tanenbaum Centre for Pharmacogenetics, which is advancing research for the CAMH Pharmacogenetic Program. We would like to thank all the subjects who generously participated in this study. We thank the Ministry of Research and Innovation of Ontario for funding the IMPACT project.

## Conflict of Interest

The authors declare that the research was conducted in the absence of any commercial or financial relationships that could be construed as a potential conflict of interest.
